# Genome-Wide Characterization of Fructose 1,6-Bisphosphate Aldolase Genes and Expression Profile Reveals Their Regulatory Role in Abiotic Stress in Cucumber

**DOI:** 10.3390/ijms25147687

**Published:** 2024-07-13

**Authors:** Jinlong Zhang, Yike Liu, Zhenpeng Zhou, Lina Yang, Zhanjun Xue, Qingyun Li, Bingbing Cai

**Affiliations:** College of Horticulture, Hebei Agricultural University, Baoding 171000, China; hope20010104@163.com (J.Z.); liuyike1024@163.com (Y.L.); cr7666999@163.com (Z.Z.); lenayang0830@163.com (L.Y.); xzj_0117@126.com (Z.X.)

**Keywords:** fructose-1,6-bisphosphate aldolase, cucumber, genome-wide identification, abiotic stresses

## Abstract

The fructose-1,6-bisphosphate aldolase (*FBA*) gene family exists in higher plants, with the genes of this family playing significant roles in plant growth and development, as well as response to abiotic stresses. However, systematic reports on the *FBA* gene family and its functions in cucumber are lacking. In this study, we identified five cucumber *FBA* genes, named *CsFBA1*-*5*, that are distributed randomly across chromosomes. Phylogenetic analyses involving these cucumber FBAs, alongside eight Arabidopsis FBA proteins and eight tomato FBA proteins, were conducted to assess their homology. The CsFBAs were grouped into two clades. We also analyzed the physicochemical properties, motif composition, and gene structure of the cucumber FBAs. This analysis highlighted differences in the physicochemical properties and revealed highly conserved domains within the CsFBA family. Additionally, to explore the evolutionary relationships of the CsFBA family further, we constructed comparative syntenic maps with *Arabidopsis* and tomato, which showed high homology but only one segmental duplication event within the cucumber genome. Expression profiles indicated that the *CsFBA* gene family is responsive to various abiotic stresses, including low temperature, heat, and salt. Taken together, the results of this study provide a theoretical foundation for understanding the evolution of and future research into the functional characterization of cucumber FBA genes during plant growth and development.

## 1. Introduction

Cucumber (*Cucumis sativus* L.) is among the most crucial vegetable crops globally, having a tropical origin. This crop is sensitive to cold and, therefore requires substantial energy for greenhouse production in cooler seasons [1]. Cucumbers are vulnerable to chilling temperatures, a major environmental stress that substantially affects their growth and development, often resulting in substantial economic losses. 

Throughout their long evolutionary history, plants have developed complex regulatory networks to detect stress signals and adapt to adverse conditions through interaction of polysaccharides and water molecules [2] or by regulating metabolic processes. Chloroplasts, which are essential for photosynthesis in green plants, enable plants to respond to environmental stresses and adapt to adverse environmental conditions by regulating metabolism pathways [3,4]. They are the sites of photosynthesis and oxygen release processes that produce energy to support plant growth and yield. Chloroplasts also synthesize essential compounds such as amino acids, phytohormones, nucleotides, vitamins, lipids, and secondary metabolites in response to environmental stresses [5]. They act as environmental sensors, relaying signals to adjust the metabolic processes and gene expressions in plants to cope with stresses [6,7,8]. Studies have shown that chloroplasts enhance photosynthesis and plant resilience under adverse environmental conditions [9]. Under extreme conditions, such as low temperatures, heat, and salinity, chloroplasts produce reactive oxygen species (ROS) that can damage the bio-membrane system and further deteriorate plant health and viability. Excessive ROS can also hinder carbon fixation by deactivating enzymes in the Calvin–Benson cycle, thereby reducing photosynthesis, which is crucial for carbohydrate synthesis. However, ROS can also be served as a feedback signal to manage stresses [9]. In response to low temperatures, the structure and metabolism of chloroplasts undergo alteration, leading to increases in the plant’s tolerance to these conditions. Adaptation to chilling stress involves regulating the activities of dark-reaction-related enzymes in chloroplasts, such as fructose-1,6-diphosphatase (FBPase) and isoheptanone-1,7-diphosphatase (SBPase) [10]. In addition, researches have confirmed that various plant hormones, such as auxin (IAA) [11], abscisic acid (ABA) [12,13,14], salicylic acid (SA) [15,16], jasmonic acid (JA) [17], and ethylene (ET) [18,19,20], as well as related genes and transcription factors, play crucial roles in regulating plants’ chilling tolerance. The transcription factor dehydration-responsive element binding factor 1 (DREB1)/C-repeat binding factor (CBF/DREB1) is particularly known for its role in cold acclimation. The overexpression of this factor in *Arabidopsis* and cucumber activates the downstream cold-responsive (COR) genes to respond to this stress [21,22,23,24,25,26,27]. 

Fructose-1,6-bisphosphate aldolase (EC 4.1.2.13, FBA) is a critical enzyme associated with both the glycolysis/gluconeogenesis pathway and the Calvin cycle. It can be divided into class I and class II FBAs. Higher plants contain mainly class I FBAs, which are present in the plastids and cytoplasm and play crucial roles in enzymatic reactions and in regulating stress tolerance [28,29]. Extensive research on *FBA* genes in various plant species has enhanced our understanding of this gene family. Different members of the *FBA* family have been identified and characterized in numerous plant species, such as *Arabidopsis* [28], tomato [29], rice [30], maize [31], spinach [32], soybean [33], potato [34], oat [35], tobacco [36], purslane [37], wheat [38], moso bamboo [39], and tea tree oil [40]. In *Arabidopsis* and tomato, the *FBA* gene family consists of eight members, named *FBA1*-*8*. However, in rice, the *FBA* family consists of only seven members, named *ALD1*-*6* and *ALDY*. These variations suggest that gene families have expanded or contracted during evolution. Studies have also explored the diverse functions of *FBA* genes in response to various biotic and abiotic stresses. Numerous studies have indicated that the *FBA* gene can respond to various stresses, such as salinity [36,37], drought [41], and temperature [35,42,43]. 

Although *FBA* genes have been extensively studied in many species, little is known regarding this gene family in the cucumber (*C. sativus* L.) genome. However, the recent publication of the cucumber genome sequence (available at http://cucurbitgenomics.org/; accessed on 8 April 2024) provides an opportunity to explore the *FBA* gene family in cucumber, as well as its evolutionary history. To address this research gap and to predict the phenotype, which is one of the central tasks of genome research [44], we used various bioinformatics methods to identify *FBA* genes in the cucumber genome and analyzed their expression levels under different abiotic stresses because the network-based method achieves more accurate predictions and better interpretability [45]. In addition, we identified some candidate genes for further functional research in the field.

In this study, we identified five *CsFBA* genes and divided them into two subgroups. We conducted phylogenetic and synteny analyses, assessed gene structures, and examined conserved motifs. In addition, we analyzed the expression profiles of *CsFBA* genes under low-temperature, heat, and salt conditions to understand how these genes respond to these abiotic stresses. 

## 2. Results

### 2.1. Genome-Wide Identification of CsFBA Genes in Cucumber

Five members of the *FBA* gene family in the cucumber genome were identified through BlastP analysis, all previously annotated as fructose phosphor aldolase. These members were designated as *CsFBA1* to *CsFBA5* based on their chromosomal locations in the official database. Specific details of these genes, including sequence ID, amino acid count (length), protein molecular weight (MW), theoretical isoelectric point (pI), instability index, aliphatic index, and grand average of hydropathicity, are presented in Table 1. Among the identified members, CsFBA1 had the most amino acids (397), whereas CsFBA5 had the fewest amino acids (357). The MW ranged from 37.97 kDa (CsFBA5) to 42.89 kDa (CsFBA1). The pI values ranged from 6.07 for CsFBA5 to 8.69 for CsFBA3, with three of these members having acidic properties. The grand average of hydropathicity indicates that all five members were hydrophilic proteins.

### 2.2. Phylogenetic Analysis of the FBA Family from Arabidopsis, Tomato, and Cucumber Genomes

**Figure 1:**
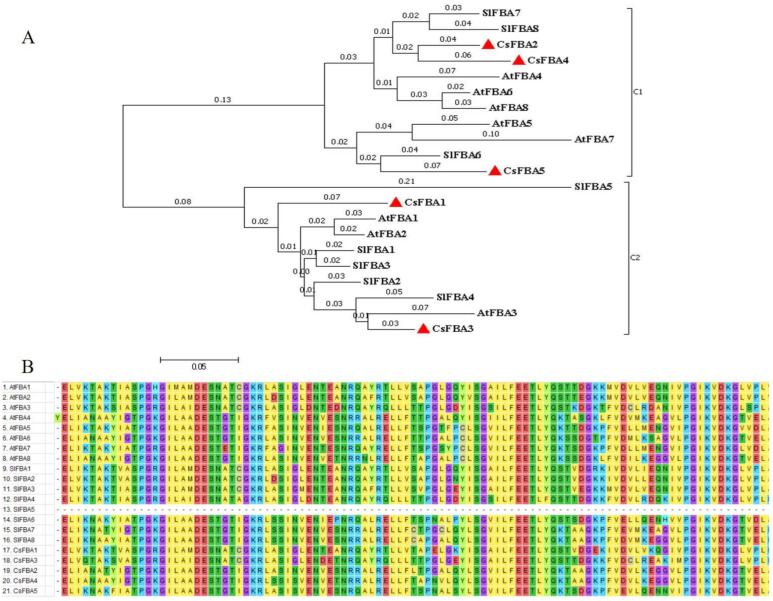
Evolutionary relationships of FBA family in cucumber, Arabidopsis, and tomato. (**A**) Phylogenetic tree of the relationship between the FBA proteins of cucumber, Arabidopsis, and tomato. C1 and C2 represent different subfamilies. The evolutionary history was inferred using the neighbor-joining method [46]. The optimal tree with a sum of branch length = 1.60678193 is shown. The tree is drawn to scale, with branch lengths (above the branches) in the same units as those of the evolutionary distances used to infer the phylogenetic tree. The evolutionary distances were computed using the p-distance method [47] and are presented as the number of amino acid differences per site. The analysis involved 21 amino acid sequences. All positions containing gaps and missing data were eliminated, resulting in a final dataset comprising a total of 132 positions. Evolutionary analyses were conducted using MEGA7.0 [48]. (**B**) Multiple sequence alignment of the glycolysis domains of 21 FBA proteins from cucumber, *A. thaliana*, and tomato using 100 amino acids on either side of the structural domain.

Based on the full-length amino acid sequences of the five cucumber FBA proteins (CsFBAs), along with eight *Arabidopsis* FBAs (AtFBAs) and eight tomato FBAs (SlFBAs) identified previously, a phylogenetic tree was constructed using MEGA 7.0 software (Figure 1A). The FBA proteins were classified into two clades. Clade 1 comprised CsFBA2, CsFBA4, and CsFBA5, together with AtFBA4-8 and SlFBA6-8, which were predicted to be located in the cytoplasm. Conversely, CsFBA1 and CsFBA3, along with AtFBA1-3 and SlFBA1-5, with their subcellular location predicted to be the chloroplast, fell into clade 2. Furthermore, the phylogenetic tree showed close clustering of CsFBA2 and CsFBA4 with SlFBA7 and CsFBA3 with SlFBA4, suggesting high homology among these proteins and potentially similar gene functions.

To further explore the evolutionary relationships and structural domains of CsFBA proteins, multiple sequence alignments of protein sequences from AtFBAs, SlFBAs, and CsFBAs were conducted (Figure 1B). Specifically, 100 amino acid sequences from the N terminal, containing the structural domains, were selected for this analysis. The results indicated that the sequences within the structural domain were highly conserved, with the exception of SlFBA5.

### 2.3. Gene Structure and Conserved Motif Analysis

**Figure 2:**
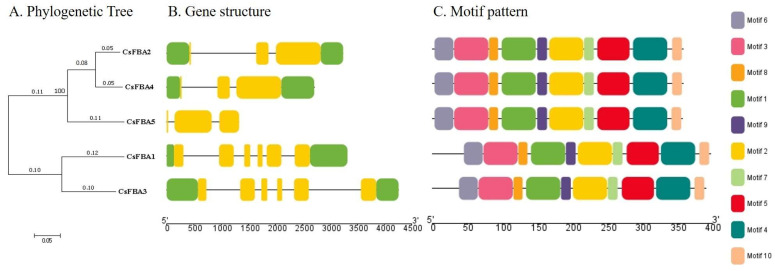
Phylogenetic tree, gene structure, and motif pattern of CsFBA proteins. (**A**) The phylogenetic tree was constructed using the full-length sequences of CsFBA proteins with 1000 replicates on each node. (**B**) Green rectangles, yellow rectangles, and black lines indicate UTRs (non-coding regions), CDSs (coding sequences or exons), and introns, respectively. (**C**) The amino acid motifs (numbered 1–10) in CsFBA proteins are displayed in ten colored boxes, and black lines indicate amino acid length.

To analyze the structural composition of *CsFBA* genes, a structural map was created using the cucumber genome sequence. This map included the untranslated regions (UTRs), coding sequences (CDSs), and introns (Figure 2A,B). A comparison of the number and placement of exons and introns across the *CsFBA* genes revealed variability; *CsFBA* genes in clade 1 (C1) had three exons each, whereas those in clade 2 (C2) contained six exons each. Notably, *CsFBA5* had no UTRs. To further explore the conservation of the *CsFBA* gene family throughout evolution, we analyzed their conserved motif patterns using TBtools. Ten conserved motifs, designated as motifs 1 to 10 and distinguished by various colors, were identified. The presence of all 10 motifs in each of the five CsFBA proteins suggested a high degree of conservation throughout evolutionary history.

### 2.4. Chromosomal Distribution and Gene Duplication Analysis of CsFBA Genes

**Figure 3:**
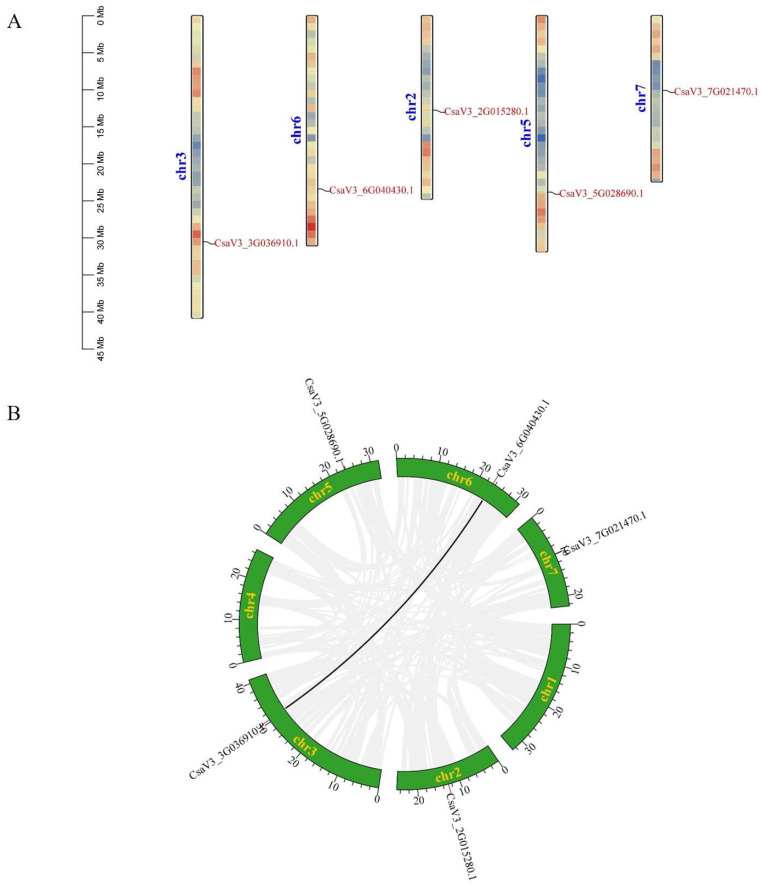
(**A**) Chromosomal location of *CsFBAs*. The colored rectangular bars represent the chromosomes of cucumber, and the 0–45 Mb scale represents chromosome length. (**B**) Synteny analysis of *FBA* genes on cucumber chromosomes. Seven cucumber chromosomes are colored in green with their names. The black line in the figure denotes the syntenic region of the cucumber genome.

The five *CsFBA* genes were located on five chromosomes, with one gene per chromosome; *CsFBA1* was on chromosome 2, *CsFBA2* on chromosome 3, *CsFBA3* on chromosome 5, *CsFBA4* on chromosome 6, and *CsFBA5* on chromosome 7 (Figure 3A). Gene duplication events, which include tandem and segmental duplications, were analyzed to understand the mechanisms underlying the expansion or contraction of the *CsFBA* gene family. A tandem duplication event is defined as the presence of two or more genes in a chromosomal region within 200 kb [49]. Our analysis revealed no tandem duplication events within the *CsFBA* family. However, one segmental duplication event was identified, involving a pair of genes (*CsFBA2* and *CsFBA4*) within the cucumber genome (Figure 3B).

### 2.5. Analysis of Collinearity and Evolutionary Relationships between CsFBA and FBA Proteins in Arabidopsis and Tomato Genomes

**Figure 4:**
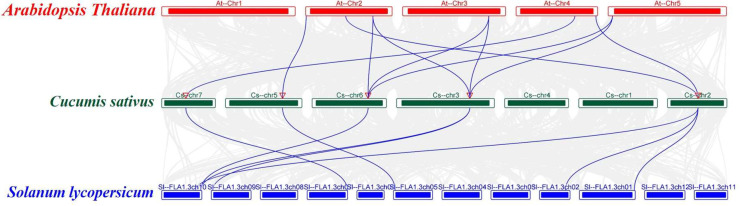
Collinear analyses of *FBA* genes between cucumber, *Arabidopsis*, and tomato. The gray lines between cucumber and other plants represent collinear blocks in wide regions of the genomes, while blue lines show the orthologous relationship of *FBA* genes.

To investigate the evolutionary relationships between the *CsFBA* gene family in cucumber and *FBA* genes in other species, a collinear map of cucumber with *Arabidopsis* and tomato was constructed (Figure 4). This analysis revealed a strong collinear relationship between the *CsFBA* gene family and the *FBA* genes of *Arabidopsis* and tomato. Specifically, the five *CsFBA* genes showed collinearity with seven *AtFBA* genes from Arabidopsis and with seven out of the eight *SlFBA* genes from tomato.

### 2.6. Expression Pattern of CsFBAs in Different Conditions

**Figure 5:**
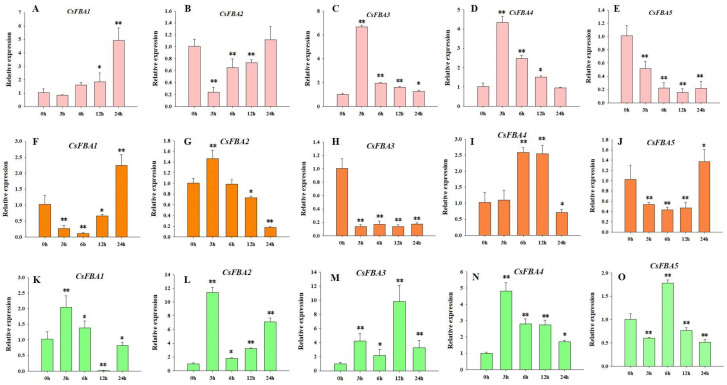
Expression patterns of the five *CsFBA* genes in cucumber seedlings after low-temperature treatment (**A**–**E**), heat treatment (**F**–**J**), and salt treatment (**K**–**O**). * Significant differences according to t-test (* *p* < 0.05, ** *p* < 0.01).

*FBA* genes play a crucial role in responding to abiotic stresses and affect plant growth and development [29]. To further explore the role of the *CsFBA* genes in response to various stresses, cucumber seedlings were subjected to low-temperature, heat, and salt stresses. The samples were collected at intervals of 3, 6, 12, and 24 h post treatment, with 0 h serving as the control. The relative expressions of the five *CsFBA* genes were measured. As shown in Figure 5, all five *CsFBA* genes responded to low-temperature, heat, and salt treatments. The expression of *CsFBA1*, *CsFBA3*, and *CsFBA4* was particularly induced by low-temperature stress, with *CsFBA3* and *CsFBA4* showing a substantial increase within the first 3 h. In contrast, the expression levels of *CsFBA2* and *CsFBA5* were suppressed under low-temperature conditions. Under heat stress, the expression of *CsFBA2* and *CsFBA4* increased, whereas the expression levels of *CsFBA1*, *CsFBA3*, and *CsFBA5* decreased. Moreover, 24 h after heat treatment, the expression levels of *CsFBA1* and *CsFBA5* recovered to their original levels or even exceeded those at the 0 h mark, suggesting a possible self-regulation mechanism in plants. All five *CsFBA* genes were also induced at various times after exposure to salt stress, indicating a broad spectrum of stress responsiveness.

## 3. Discussion

Many gene families play essential roles in metabolic and development processes [50]. In recent years, the focus on understanding regulatory mechanisms has led to the identification of many gene families. FBAs are widely distributed in various higher plant species and are essential for regulating plant resistance to biotic and abiotic stresses [49]. To date, information on the *FBA* gene family has been reported in *Arabidopsis* [28], tomato [29], and rice genomes [40], with particular attention to the structure, kinetic parameters, and potential therapeutic applications of these genes. However, reports on the *FBA* gene family in the cucumber genome are lacking, and there has been limited study on the functions of this gene family. Are they regulatory factors in plant growth and tolerance to abiotic stresses? Are they candidate genes for the breeding of new varieties of vegetables with low-temperature tolerance? In this study, we used various bioinformatics methods to identify the *FBA* gene family in cucumber. Our objectives were to (1) identify the *FBA* gene family in the cucumber genome, (2) predict the responses of genes to abiotic stresses, and (3) select some candidate *CsFBA* genes for further functional research on regulating growth or metabolic pathways in plants.

In this study, we identified five *CsFBA* genes in cucumber, which contains fewer FBA members than *Arabidopsis*, tomato, and rice. This suggests that the gene family may have undergone contraction during evolution, potentially leading to the loss of some functionalities. We also analyzed the physicochemical properties of these proteins, including amino acid count (length), protein MW, pI, instability index, aliphatic index, and grand average of hydropathicity (Table 1). The variations among the five *CsFBA* genes indicate divergence in the evolutionary paths due to changing environmental conditions. Phylogenetic and subcellular localization prediction analyses suggested that CsFBA1 and CsFBA3 are homologous with chloroplast/plastid FBAs, such as AtFBA1-3 and SlFBA1-4, whereas CsFBA2, CsFBA4, and CsFBA5 are similar to cytosolic FBAs, such as AtFBA4-8 and SlFBA5-8 (Figure 1A) [28,29]. Chloroplast is the organelle for photosynthesis, and the *FBA* genes in this clade play a crucial part in both photosynthetic and non-photosynthetic metabolism pathways and, therefore, in regulating development and tolerance to low-temperature stress [51]. A previous study showed that over-expression of *SlFBA7* in tomato led to an enhanced net photosynthetic rate and activity of other enzymes in the Calvin cycle. However, a study of mutants in *Arabidopsis* showed that there is functional redundancy of *AtFBA1*, *AtFBA2*, and *AtFBA3* in regulating plant growth. Because the *fba1* mutants showed no phenotype distinguishable from wild type, while both the *fba2* and *fba3* mutants showed reduced growth, double mutants *fba2/fba3* were lethal [51]. This provides a bright insight in the study of gene family functions. The cytolist-located FBA gene *AtFBA6* not only functions in metabolic process but also works as a transcription factor [52]. It has a relatively higher expression level in shoot-apical meristem, root-apical meristem, and the vascular bundle, and the protein can interact with WUS, WOX4, and WOX5 [52]. For protein function studies, SDS-PAGE and in vitro enzymatic synthesis of biochemicals can provide more bright ideas [53]. This differentiation in localization suggests varied functions among the members, with plastid-located proteins CsFBA1 and CsFBA3 likely playing crucial roles in the Calvin cycle, particularly in response to low-temperature stress. The conserved gene structure and motifs across these genes imply functional conservation, with orthologous genes typically sharing similar functions and grouped within the same phylogenetic clades. The collinear relationship of *CsFBA* genes with those of *Arabidopsis* and tomato provides insights into the potential functions of cucumber *FBA* genes. The results of collinearity analysis (Figure 4) align with previous studies suggesting that all *AtFBA* genes play a role in response to abnormal temperature stresses. Phylogenetic studies suggest that *CsFBA2*, *CsFBA3*, and *CsFBA4* share a high homology with *SlFBA7*, further indicating the specific roles of these genes in responding to low-temperature stress, warranting further investigation. In addition, we found that all five *CsFBA* genes respond to low-temperature, heat, and salt stresses to different degrees. Notably, *CsFBA1*, *CsFBA3*, and *CsFBA4* were induced by low-temperature and salt stresses (Figure 5), indicating their similar roles in response to low-temperature and salt stresses. However, under heat stress, the expression of *CsFBA1*, *CsFBA3*, and *CsFBA5* was suppressed, while the remaining genes were induced. These results indicate that these genes regulate stress tolerance, possibly through different mechanisms. The plastid, a key site for photosynthesis and biosynthesis of starch and sugar, plays a significant role. In *Arabidopsis*, the *fba2* mutant displayed growth retardation under short-day conditions, unlike the *fba1* mutant, which grew normally, suggesting a more pivotal role of *AtFBA2* in regulating plant growth [51]. Similarly, the *fba8* mutant exhibited a distinct phenotype compared with the wide type. Based on these observations, we hypothesize that *CsFBA* genes may be involved in regulating plant growth and development, warranting further explorations.

## 4. Materials and Methods

### 4.1. Plant Materials, Growth Conditions, and Treatments

We used cucumber cultivar ‘Jinyou 35’, a prickly Chinese long cucumber. The seeds were soaked in distilled water for 4–6 h, then sown on moist filter paper, allowing them to germinate in the dark at 28 °C. After three days of germination, the seeds were transferred to nutrition pots filled with culture substrate and placed in a solar greenhouse. Here, they received natural sunlight during the day (with a peak photosynthetic photon flux density of 800–1000 μmol/m²/s) and were maintained at a day/night temperature of 25 °C–31 °C/13 °C–21 °C under a 13 h photoperiod [54]. Once the seedlings developed three true leaves, they were subjected to different stress treatments. For low-temperature and heat stress treatments, the seedlings were moved to growth chambers set at 8 °C/5 °C (day/night) and 42 °C/32 °C (day/night), respectively. The second leaves were harvested 0, 3, 6, 12, and 24 h after initiating treatment; immediately frozen in liquid nitrogen; and stored at −80 °C for subsequent analysis. For salt stress, the seedlings were treated with Okazaki nutrient solution adjusted to a salinity concentration of 150 mmol/L. The second leaves were harvested at different time intervals similar to those in the low-temperature and heat stress treatments, quickly frozen in liquid nitrogen, and stored at −80 °C for analysis.

### 4.2. Identification of CsFBA Genes in the Cucumber Genome

Arabidopsis FBA protein sequences were used as a query and searched against the cucumber genome database (http://cucurbitgenomics.org/; accessed on 8 April 2024). The GFF3 file of the cucumber genome database was downloaded from the Cucurbit Genomics Database. Sequence analysis was conducted using the Local BlastP program through TBtools. Pfam (http://pfam.janelia.org; accessed on 8 April 2024) and SMART (http://smart.embl-heidel-berg.de/; accessed on 8 April 2024) tools were used to confirm the glycolytic domain (PF00274) and verify the reliability of each candidate CsFBA protein as a cucumber FBA family member.

### 4.3. Phylogenetic Analysis

Protein sequences for *Arabidopsis*, tomato, and cucumber were prepared in FASTA format. Multiple sequence alignments were performed using ClustalX 1.8, with default parameters. The phylogenetic tree was constructed using MEGA 7.0 [48]. The neighbor-joining method [46] was used with the following parameters: p-distance substitution model [47], pairwise deletion, and bootstrap (1000 replicates; random seed) [55].

### 4.4. Gene Structure and Conserved Motif Analysis

For the analysis of the cucumber *FBA* gene family and multiple sequence comparisons, we performed structural mapping of *CsFBA* genes and visualized conserved motifs by using TBtools-II v2.096 software, utilizing a previously prepared GFF3 file. The search for maximum conserved motifs was set to 10, with all other parameters left at their default settings.

### 4.5. Chromosomal Distribution and Synteny Analysis of CsFBA Genes

Genomic FASTA files and genome annotation GFF3 files were prepared to analyze the chromosomal distribution of the genes. These files were then input into the One Step MSCanX program within TBtools-II v2.096. This process highlighted target genes on the visual map, providing a clear representation of their chromosomal locations.

### 4.6. Total RNA Extraction, Reverse Transcription, and qRT-PCR Analysis

Samples from Section 2.1 (weighing 0.1 g) were thoroughly ground in liquid nitrogen, and total RNA was extracted using TRIzol reagent as per the manufacturer’s instructions. The integrity of RNA was assessed using 1% agarose gel electrophoresis, whereas its purity and concentration were measured using a spectrophotometer. For cDNA synthesis, 1 μg of high-quality total RNA was used. Specific primers for quantitative real-time PCR (qRT-PCR) were designed using Primer Premier software (version 5.0; Premier, Nanaimo BC, Canada). These primers were selected to prevent the amplification of conserved regions and exclusively amplify products ranging from 150 to 300 bp in length. β-actin served as an internal control for the experiments. Quantitative real-time PCR was conducted using PerfecStart Green qPCR Super Mix, following the manufacturer’s protocol. The amplification was conducted on a BIO-RAD CFX96 real-time PCR detection system (Bio-Rad, Hercules, CA, USA). Each sample type was analyzed in triplicate. The relative mRNA expression levels of the target genes were calculated using the 2^−∆∆Ct^ method [56].

### 4.7. Statistical Analysis

For this study, data entry and statistical analyses were conducted using Microsoft Excel 2010. Statistical differences were determined using DPS 9.01 software, employing Student’s *t*-test. Bar graphs were generated using Origin 2020. Statistical significance was determined at two levels, namely *p* < 0.05 and *p* < 0.01, which are denoted by asterisks (*) and double asterisks (**), respectively, in the figures.

## 5. Conclusions

Our study systematically identified five *CsFBA* genes from the whole genome of cucumber. The physicochemical properties of the *CsFBA* genes are similar, and their structures are conserved. The five *CsFBA* genes are distributed across five chromosomes, with only one pair of genes derived from segmental duplication. Moreover, the *CsFBA* gene family responds to temperature and salt stresses, indicating their potential involvement in environmental stress responses. This study not only provides a theoretical basis but also lays the basis for future research into the functions and mechanisms of cucumber *FBA* genes in plant development and responses to abiotic stresses.

## Figures and Tables

**Table 1:** Summary information of *FBA* gene family in cucumber.

Gene Name	Sequence ID	Number of Amino Acids	Molecular Weight (KD)	Theoretical pI	Instability Index	Aliphatic Index	Grand Average of Hydropathicity
CsFBA1	CsaV3_2G015280	397	42.89	6.39	33.92	92.42	−0.126
CsFBA3	CsaV3_5G028690	390	42.38	8.69	41.65	84.87	−0.247
CsFBA2	CsaV3_3G036910	358	38.60	6.49	29.59	90.84	−0.199
CsFBA4	CsaV3_6G040430	358	38.41	7.57	28.19	94.08	−0.164
CsFBA5	CsaV3_7G021470	357	37.97	6.07	34.28	91.6	−0.093

## Data Availability

All data presented in the study are included in the article. Further inquiries can be directed to the corresponding author.

## Abbreviations

FBA	Fructose-1, 6-bisphosphate aldolase
ROS	Reactive oxygen species
FBPase	Fructose-1,6-diphosphatase
SBPase	Isoheptanone-1,7-diphosphatase
IAA	Indoleacetic acid
ABA	Abscisic acid
SA	Salicylic acid
JA	Jasmonic acid
ET	Ethylene
DREB1	Dehydration-responsive element binding factor 1
CBF	C-repeat binding factor
COR	Cold response
qRT-PCR	Quantitative real-time PCR
MW	Molecular weight
pI	Isoelectric point
